# Data in support of the comparative genome analysis of *Lysinibacillus* B1-CDA, a bacterium that accumulates arsenics

**DOI:** 10.1016/j.dib.2015.09.040

**Published:** 2015-10-09

**Authors:** Aminur Rahman, Noor Nahar, Neelu N. Nawani, Jana Jass, Sibdas Ghosh, Björn Olsson, Abul Mandal

**Affiliations:** a Systems Biology Research Center, School of Bioscience, University of Skövde, P.O. Box 408, SE-541 28 Skövde, Sweden; b Dr. D. Y. Patil Biotechnology and Bioinformatics Institute, Dr. D. Y. Patil Vidyapeeth, Tathawade, Pune 411033, India; c The Life Science Center, School of Science and Technology, Örebro University, SE-701 82 Örebro, Sweden; d School of Arts and Science, Iona College, New Rochelle, NY 10801, USA

## Abstract

This study is a part of our long term project on bioremediation of toxic metals and other pollutants for protection of human health and the environment from severe contamination. The information and results presented in this data article are based on both *in vitro* and *in silico* experiments. *in vitro* experiments were used to investigate the presence of arsenic responsive genes in a bacterial strain B1-CDA that is highly resistant to arsenics. However, *in silico* studies were used to annotate the function of the metal responsive genes. By using this combined study consisting of *in vitro* and *in silico* experiments we have identified and characterized specific genes from B1-CDA that can be used as a potential tool for removal of arsenics as well as other heavy metals from the contaminated environment.

**Specifications table**

**Table:** 

Subject area	Biology
More specific subject area	Molecular biology, Microbiology. Studies of arsenic responsive genes as well as other metal responsive genes in bacteria
Type of data	Tables and figure
How data was acquired	The data was derived by NGS as a raw data then *de novo* assembly and gene annotation was performed
Data format	Analyzed
Experimental factors	Bacterial isolate *Lysinibacillus sphaericus* B1-CDA was cultured in the presence of 100 mM arsenate and then DNA was isolated from these cells
Experimental features	Genome sequencing and annotation of metal responsive genes in *L. sphaericus* B1-CDA
Data source location	Bacterial sample was collected from a highly arsenic-contaminated cultivated land located in the south-west region of Bangladesh. DNA analysis was performed at the University of Skövde, Sweden and NGS and *de novo* assembly at Otogenetics Corporation in Norcross, USA
Data accessibility	The genome information is available in EMBL as follows: [GenBank accession number LJYY01000000, http://www.ncbi.nlm.nih.gov/nuccore/LJYY00000000]

**Value of the data**

• Complete genome sequencing of a highly arsenic resistant bacteria *L*. *sphaericus*, strain B1-CDA.
• Annotation of bacterial genes involved in binding and transport of toxic metals such as arsenics.
• Data presented in this article can be used to remove toxic metals from the contaminated sources thus protecting human health and the environment.
• In a longer term these data can also contribute to socio-economic development of a society.

## 1 Data

The information and results presented in this data article are derived from the *in vitro* experiments for investigation of the arsenic responsive genes. We also provide *in silico* data on gene annotation that can be potentially useful for conducting microbial bioremediation of toxic metals.

## 2 Experimental design, materials and methods

*Lysinibacillus sphaericus* B1-CDA strain was collected from a highly arsenic-contaminated region located in the south-west region of Bangladesh. Previously, we have reported that the strain *L*. *sphaericus* B1-CDA is highly resistant to arsenic and it accumulates arsenic inside the cells [1]. Genomic DNA was extracted from this bacterium, using Master pure™ Gram positive DNA purification kit (Epicenter, USA). Genome sequencing of the strain was performed by the Otogenetics Corporation (GA, USA). After sequencing the genome was assembled by *de novo* assembly employing SOAPDenovo, version 2.04 [2].

The assembled genome sequence was annotated with Rapid Annotations using Subsystems Technology, RAST [3]. Functional annotation analysis was also carried out by the Blast2GO pipeline [4] using all translated protein coding sequences resulting from the GeneMark. An InterPro scan [5] was performed through the Blast2GO interface and the InterPro IDs were merged with the Blast-derived GO-annotation for obtaining the integrated annotation results. The GO annotation of all putative metal responsive genes was manually curated. The functional annotation carried out by the RAST and Blast2GO indicates that B1-CDA contains many genes which are responsive to specific metal ions like arsenic, cobalt, copper, iron, nickel, potassium, manganese and zinc. Prediction by RAST and Blast2GO (Table 1) revealed that the B1-CDA genome contains additionally a total of 123 proteins involved in binding and transport of metal ions. Further, B1-CDA contains many other proteins (approximately 30) that catalyze binding and transport of the metal ions such as metalloendopeptidase, metalloexopeptidase, metallopeptidase, metallocarboxypeptidase and metallochaperone (Table 2).

**Fig. 1:**
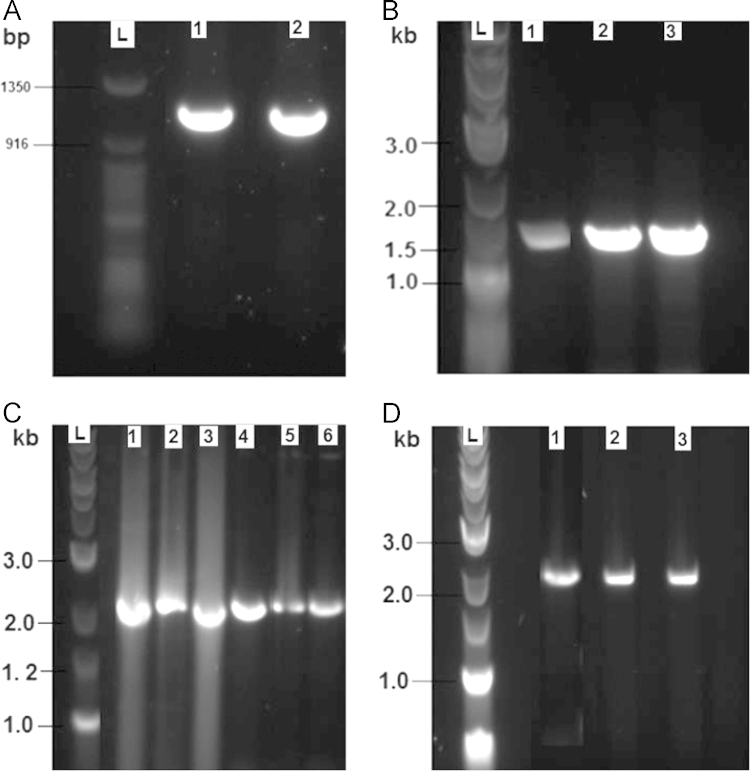
Molecular analysis of arsenic responsive genes of B1-CDA and gel electrophoresis: (A) PCR amplification of *acr3* gene. L represents 50 bp DNA marker, whereas lane 1 and 2 are the amplified fragments of *acr3* gene in two replicates. (B) PCR amplification of *arsR* gene. L represents 2-log DNA marker, whereas lane 1, 2 and 3 are the amplified fragments of *arsR* gene in three replicates. (C) PCR amplification of *arsB gene.* L represents 2-log DNA marker, whereas lane 1, 2, 3, 4, 5 and 6 are the amplified fragments of *arsB* gene in six replicates and (D) PCR amplification of *arsC* gene. L represents 2-log DNA marker, whereas lane 1, 2 and 3 are the amplified fragments of *arsC* gene in three replicates.

In this article, we have studied the presence of arsenic resistance genes in this bacterium by using PCR amplification. The strain B1-CDA was found to harbor *acr3*, *arsR*, *arsB* and *arsC* arsenic marker genes (Fig. 1). The *arsC* gene codes for the enzyme arsenate reductase, which is responsible for the biotransformation of arsenate [As(V)] to arsenite [As(III)] prior to efflux. ArsB, an integral membrane protein that pumps arsenite out of the cell, is often associated with an ATPase subunit, arsA [6]. It is hypothesized that the *arsB*/*acr3* genes are the primary determinants in arsenite resistance [6]. The results of these studies could be used to cope with arsenic toxicity by removing it from the contaminated source or converting it to a less toxic harmless compound.

## Figures and Tables

**Table 1:** Genes involved in metal ion binding and metal ion transport in B1-CDA predicted by RAST and/or Blast2GO.

Seq. name	No. of nucleotide	Start	End	Function
Gene 7	597	5406	6002	Metal ion binding
Gene 33	1464	34,171	35,634	Metal ion binding
Gene 46	1977	45,923	47,899	Metal ion binding
Gene 77	768	79,288	80,055	Metal ion binding
Gene 117	1995	127,168	129,162	Metal ion binding
Gene 171	1125	184,513	185,637	Metal ion binding
Gene 177	1425	190,919	192,343	Metal ion binding
Gene 188	2004	204,045	206,048	Metal ion binding
Gene 223	876	242,015	242,890	Metal ion binding
Gene 226	1674	244,232	245,905	Metal ion binding
Gene 238	1233	254,866	256,098	Metal ion binding
Gene 310	1587	328,134	329,720	Metal ion binding
Gene 313	1131	331,292	332,422	Metal ion binding
Gene 353	936	369,982	370,917	Metal ion binding
Gene 366	921	381,327	382,247	Metal ion binding
Gene 507	849	521,560	522,408	Metal ion binding
Gene 541	987	552,963	553,949	Metal ion binding
Gene 576	1110	596,085	597,194	Metal ion binding
Gene 578	918	598,123	599,040	Metal ion binding
Gene 600	741	615,128	615,868	Metal ion binding
Gene 603	1716	617,978	619,693	Metal ion binding
Gene 637	1164	651,099	652,262	Metal ion binding
Gene 765	1506	798,894	800,399	Metal ion binding
Gene 835	1047	884,978	886,024	Metal ion binding
Gene 845	1173	896,350	897,522	Metal ion binding
Gene 866	639	913,115	913,753	Metal ion binding
Gene 877	552	926,277	926,828	Metal ion binding
Gene 885	1113	935,597	936,709	Metal ion binding
Gene 953	1359	1,012,868	1,014,226	Metal ion binding
Gene 1010	669	1,072,202	1,072,870	Metal ion binding
Gene 1011	885	1,072,873	1,073,757	Metal ion binding
Gene 1014	1143	1,076,516	1,077,658	Metal ion binding
Gene 1039	774	1,105,712	1,106,485	Metal ion binding
Gene 1109	3435	1,174,450	1,177,884	Metal ion binding
Gene 1147	1668	1,212,657	1,214,324	Metal ion binding
Gene 1185	1185	1,255,829	1,257,013	Metal ion binding
Gene 1206	1740	1,277,562	1,279,301	Metal ion binding
Gene 1218	2067	1,294,077	1,296,143	Metal ion binding
Gene 1244	795	1,319,141	1,319,935	Metal ion binding
Gene 1271	3510	1,348,576	1,352,085	Metal ion binding
Gene 1294	2511	1,372,937	1,375,447	Metal ion binding
Gene 1322	2364	1,399,790	1,402,153	Metal ion binding
Gene 1389	204	1,481,600	1,481,803	Metal ion binding
Gene 1397	1002	1,491,472	1,492,473	Metal ion binding
Gene 1408	3249	1,500,339	1,503,587	Metal ion binding
Gene 1426	873	1,523,169	1,524,041	Metal ion binding
Gene 1432	1308	1,534,294	1,535,601	Metal ion binding
Gene 1467	765	1,563,504	1,564,268	Metal ion binding
Gene 1485	2079	1,582,041	1,584,119	Metal ion binding
Gene 1488	546	1,586,661	1,587,206	Metal ion binding
Gene 1536	1146	1,627,858	1,629,003	Metal ion binding
Gene 1564	1668	1,656,799	1,658,466	Metal ion binding
Gene 1571	1272	1,667,080	1,668,351	Metal ion binding
Gene 1572	1302	1,668,348	1,669,649	Metal ion binding
Gene 1580	1560	1,676,269	1,677,828	Metal ion binding
Gene 1612	1269	1,708,512	1,709,780	Metal ion binding
Gene 1667	1995	1,762,661	1,764,655	Metal ion binding
Gene 1684	558	1,776,672	1,777,229	Metal ion binding
Gene 1799	906	1,887,495	1,888,400	Metal ion binding
Gene 1874	417	1,959,673	1,960,089	Metal ion binding
Gene 1889	507	1,974,321	1,974,827	Metal ion binding
Gene 1905	837	1,986,794	1,987,630	Metal ion binding
Gene 1925	1263	2,011,440	2,012,702	Metal ion binding
Gene 2028	1203	2,120,007	2,121,209	Metal ion binding
Gene 2082	615	2,173,172	2,173,786	Metal ion binding
Gene 2132	1428	2,225,179	2,226,606	Metal ion binding
Gene 2177	1212	2,268,158	2,269,369	Metal ion binding
Gene 2223	1161	2,313,715	2,314,875	Metal ion binding
Gene 2227	1713	2,316,637	2,318,349	Metal ion binding
Gene 2450	504	2,494,701	2,495,204	Metal ion binding
Gene 2477	237	2,515,822	2,516,058	Metal ion binding
Gene 2624	984	2,622,804	2,623,787	Metal ion binding
Gene 2635	1917	2,628,954	2,630,870	Metal ion binding
Gene 2859	969	2,848,824	2,849,792	Metal ion binding
Gene 3007	2706	2,987,030	2,989,735	Metal ion binding
Gene 3035	459	3,008,209	3,008,667	Metal ion binding
Gene 3036	366	3,008,700	3,009,065	Metal ion binding
Gene 3216	2607	3,159,474	3,162,080	Metal ion binding
Gene 3250	591	3,187,349	3,187,939	Metal ion binding
Gene 3252	1017	3,188,465	3,189,481	Metal ion binding
Gene 3296	909	3,231,270	3,232,178	Metal ion binding
Gene 3300	1746	3,235,892	3,237,637	Metal ion binding
Gene 3323	1173	3,258,173	3,259,345	Metal ion binding
Gene 3331	1410	3,268,181	3,269,590	Metal ion binding
Gene 3337	1671	3,276,300	3,277,970	Metal ion binding
Gene 3393	963	3,326,901	3,327,863	Metal ion binding
Gene 3437	414	3,376,060	3,376,473	Metal ion binding
Gene 3441	2154	3,379,579	3,381,732	Metal ion binding
Gene 3442	1692	3,381,729	3,383,420	Metal ion binding
Gene 3468	1368	3,410,115	3,411,482	Metal ion binding
Gene 3576	1104	3,514,691	3,515,794	Metal ion binding
Gene 3590	1716	3,530,171	3,531,886	Metal ion binding
Gene 3654	1227	3,602,057	3,603,283	Metal ion binding
Gene 3660	615	3,606,978	3,607,592	Metal ion binding
Gene 3680	1206	3,634,474	3,635,679	Metal ion binding
Gene 3702	1116	3,656,386	3,657,501	Metal ion binding
Gene 3711	957	3,667,171	3,668,127	Metal ion binding
Gene 3712	960	3,668,449	3,669,408	Metal ion binding
Gene 3738	1932	3,693,364	3,695,295	Metal ion binding
Gene 3797	594	3,750,078	3,750,671	Metal ion binding
Gene 3857	981	3,810,517	3,811,497	Metal ion binding
Gene 3889	1146	3,839,858	3,841,003	Metal ion binding
Gene 3908	915	3,862,117	3,863,031	Metal ion binding
Gene 3964	573	3,917,646	3,918,218	Metal ion binding
Gene 4020	570	3,969,387	3,969,956	Metal ion binding
Gene 4030	2121	3,978,548	3,980,668	Metal ion binding
Gene 4038	1731	3,986,132	3,987,862	Metal ion binding
Gene 4058	969	4,006,280	4,007,248	Metal ion binding
Gene 4070	906	4,023,891	4,024,796	Metal ion binding
Gene 4215	1071	4,157,550	4,158,620	Metal ion binding
Gene 4218	1635	4,160,134	4,161,768	Metal ion binding
Gene 4272	1107	4,218,954	4,220,060	Metal ion binding
Gene 4295	1380	4,241,915	4,243,294	Metal ion binding
Gene 4298	2361	4,245,236	4,247,596	Metal ion binding
Gene 4306	2124	4,254,484	4,256,607	Metal ion transport
Gene 4346	1083	4,294,106	4,295,188	Metal ion binding
Gene 4357	1386	4,307,321	4,308,706	Metal ion binding
Gene 4400	729	4,354,264	4,354,992	Metal ion binding
Gene 4454	957	4,410,503	4,411,459	Metal ion binding
Gene 4490	1377	4,445,126	4,446,502	Metal ion binding
Gene 4495	1461	4,450,541	4,452,001	Metal ion binding
Gene 4542	654	4,490,448	4,491,101	Metal ion binding

**Table 2:** Genes involved in metalloendopeptidase, metalloexopeptidase, metallopeptidase, metallochaperone and metallocarboxypeptidase protein predicted by RAST and Blast2GO are present in B1-CDA.

Seq. name	No. of nucleotide	Start	End	Function
Gene 75	2028	76,022	78,049	Metalloendopeptidase activity
Gene 90	1065	92,201	93,265	Metalloexopeptidase activity
Gene 95	1017	96,135	97,151	Metalloendopeptidase activity
Gene 248	756	266,333	267,088	Metalloexopeptidase activity
Gene 435	675	456,865	457,539	Metallopeptidase activity
Gene 597	1509	612,142	613,650	Metalloexopeptidase activity
Gene 890	1230	940,132	941,361	Metallopeptidase activity
Gene 1251	1818	1,324,367	1,326,184	Metalloendopeptidase activity
Gene 1537	1263	1,629,050	1,630,312	Metalloendopeptidase activity
Gene 1553	1224	1,648,658	1,649,881	Metalloendopeptidase activity
Gene 1825	1287	1,914,934	1,916,220	Metalloendopeptidase activity
Gene 2009	1497	2,101,832	2,103,328	Metallocarboxypeptidase activity
Gene 2062	1815	2,151,841	2,153,655	Metalloendopeptidase activity
Gene 2087	1233	2,180,574	2,181,806	Metallopeptidase activity
Gene 2153	732	2,244,087	2,244,818	Metallopeptidase activity
Gene 2442	555	2,489,739	2,490,293	Metallopeptidase activity
Gene 2665	630	2,656,743	2,657,372	Metallochaperone activity
Gene 3223	1089	3,166,498	3,167,586	Metalloexopeptidase activity
Gene 3434	1116	3,372,197	3,373,312	Metallopeptidase activity
Gene 3478	1062	3,416,770	3,417,831	Metalloexopeptidase activity
Gene 3587	927	3,526,686	3,527,612	Metalloexopeptidase activity
Gene 3609	1269	3,548,363	3,549,631	Metallopeptidase activity
Gene 3703	1089	3,657,553	3,658,641	Metalloexopeptidase activity
Gene 3874	612	3,828,598	3,829,209	Metalloendopeptidase activity
Gene 3973	1017	3,924,998	3,926,014	Metalloendopeptidase activity
Gene 4031	474	3,980,677	3,981,150	Metalloendopeptidase activity
Gene 4110	1212	4,067,537	4,068,748	Metallopeptidase activity
Gene 4255	1698	4,199,161	4,200,858	Metalloendopeptidase activity
Gene 4381	1461	4,331,031	4,332,491	Metallopeptidase activity
Gene 4433	1191	4,384,176	4,385,366	Metallocarboxypeptidase activity

## References

[1] Rahman A., Nahar N., Nawani N.N., Jass J., Desale P., Kapadnis B.P., Hossain K., Saha A.K., Ghosh S., Olsson B., Mandal A. (2014). Isolation of a *Lysinibacillus* strain B1-CDA showing potentials for arsenic bioremediation. J Environ. Sci. Health Part A.

[2] Li R., Zhu H., Ruan J., Qian W., Fang X., Shi Z., Li Y., Li S., Shan G., Kristiansen K., Li S., Yang H., Wang J., Wang J. (2010). De novo assembly of human genomes with massively parallel short read sequencing. Genome Res..

[3] Aziz R.K., Bartels D., Best A.A., DeJongh M., Disz T., Edwards R.A., Formsma K., Gerdes S., Glass E.M., Kubal M., Meyer F., Olsen G.J., Olson R., Osterman A.L., Overbeek R.A., McNeil L.K., Paarmann D., Paczian T., Parrello B., Pusch G.D., Reich C., Stevens R., Vassieva O., Vonstein V., Wilke A., Zagnitko O. (2008). The RAST server: rapid annotations using subsystems technology. BMC Genom..

[4] Götz S., García-Gómez J.M., Terol J., Williams T.D., Nagaraj S.H., Nueda M.J., Robles M., Talón M., Dopazo J., Conesa A. (2008). High-throughput functional annotation and data mining with the Blast2GO suite. Nucleic Acids Res..

[5] Zdobnov E.M., Apweiler R. (2001). InterProScan—an integration platform for the signature-recognition methods in InterPro. Bioinformatics.

[6] Achour A.R., Bauda P., Billard P. (2007). Diversity of arsenite transporter genes from arsenic-resistant soil bacteria. Res. Microbiol..

